# Unveiling the mechanism of calcitriol in treating type 2 diabetes mellitus: A combined network pharmacology and in vitro approach targeting ERS-related pathways

**DOI:** 10.1371/journal.pone.0347246

**Published:** 2026-04-16

**Authors:** Fanqiang Zeng, Yimei Liao, Jianping Jiang, Chengjian Zhao, Shuihua Peng, Huijie Zhang, Hui Zou, Shudan Tan, Liya Qiao, Fan Liu, Juan Huo, Zhigang Yan, Yongli Xu

**Affiliations:** 1 Department of Pharmacy, Guigang City People’s Hospital, The Eighth Affiliated Hospital of Guangxi Medical University, Guigang, Guangxi, China; 2 Guangxi Key Laboratory of High-Quality Formation and Utilization of Dao-di Herbs, Guangxi Botanical Garden of Medicinal Plants, Nanning, Guangxi, China; 3 Guangxi Key Laboratory of Standardized Breeding of Medicinal Animals, Guangxi Botanical Garden of Medicinal Plants, Nanning, Guangxi, China; Universitas Muhammadiyah Aceh, INDONESIA

## Abstract

Type 2 diabetes mellitus (T2DM) has emerged as a major global public health concern, characterized by increasing prevalence and serious complications that contribute substantially to societal healthcare costs. Concurrently, endoplasmic reticulum stress (ERS) has been increasingly appreciated as a central pathogenic mechanism underpinning the development and progression of T2DM. Calcitriol has been shown to have a beneficial effect in the treatment of T2DM. However, the therapeutic targets and potential mechanisms of calcitriol against T2DM through modulated ERS-related pathways remain poorly understood. To systematically investigate these mechanisms, we adopted an integrated approach combining network pharmacology, molecular docking and experimental validation. Initial network pharmacology analysis identified STAT3, HSP90AA1, MAPK1, and PIK3R1 as potential key targets mediating calcitriol’s anti-T2DM effects. These computational predictions were then experimentally validated using a high glucose (HG)-induced MIN6 cell model. Functional assessments using CCK-8 assays and flow cytometry demonstrated that calcitriol improved the survival of pancreatic β-cells and reduced glucose-induced cell death. Moreover, the results from real-time fluorescent quantitative PCR and western blot analysis revealed that calcitriol reversed the HG induced upregulation of STAT3, HSP90AA1, MAPK1 and PIK3R1 mRNA and protein levels in MIN6 cells. The findings from our study may offer insights into the underlying mechanisms through which calcitriol exerts its therapeutic effects on T2DM by targeting potential ERS-related pathways.

## Introduction

Diabetes mellitus (DM) is a chronic metabolic disorder characterized by persistent hyperglycemia, posing a pressing global health challenge. The disease confers a substantial burden, including devastating complications, significant disability, reduced life expectancy, and enormous healthcare costs [1]. Recent epidemiological data from the International Diabetes Federation (2021) reveals a staggering 537 million affected adults (aged 20–79) worldwide, with projections indicating this number will surge to 780 million by 2045 [2]. Type 2 diabetes mellitus (T2DM) constitutes 90–95% of all DM cases and has emerged as the third most threatening chronic disease to human health, following only cancer and cardiovascular diseases [3]. The clinical burden of T2DM is substantial and growing, a crisis compounded by its progressive nature, escalating global incidence, and life-altering complications such as vision loss, end-stage renal disease, and major cardiovascular events [4]. This growing epidemic underscores the critical need for effective prevention and management strategies to curb both the onset and progression of T2DM worldwide.

The pathogenesis of T2DM is underpinned by two fundamental defects: pancreatic β-cell dysfunction resulting in impaired insulin secretion, and peripheral insulin resistance (IR) in target tissues [5]. These core defects drive systemic metabolic disturbances that disrupt carbohydrate, lipid, and protein homeostasis [6]. During the initial phase of T2DM, moderate hyperglycemia and peripheral IR trigger compensatory hyperinsulinemia through enhanced β-cell insulin synthesis and secretion, temporarily maintaining glucose homeostasis [7]. However, persistent metabolic stressors such as endoplasmic reticulum stress (ERS), oxidative stress, and mitochondrial dysfunction gradually diminish the ability of pancreatic β-cells to compensate for this stress, leading to unimpeded development of T2DM. Growing evidence highlights ERS as a central mediator in T2DM pathogenesis, contributing significantly to both β-cell failure and IR development [3,8,9]. Mechanistically, ERS has been shown to disrupt multiple metabolic pathways, inducing insulin resistance, impairing islet function, and dysregulating both glucose and lipid metabolism [10]. These findings collectively position ERS as a promising therapeutic target for T2DM intervention.

Vitamin D, an essential fat-soluble micronutrient vital for maintaining human health, has demonstrated significant associations with various disease states. As a widely utilized nutritional supplement globally, vitamin D plays a therapeutic role in multiple pathological conditions [11]. Notably, accumulating evidence indicates that vitamin D status is closely associated with T2DM risk, disease progression, and the development of IR [12,13]. Vitamin D activation follows a defined metabolic pathway, requiring sequential hydroxylation in the liver and kidneys, culminating in the production of its biologically active form. Evidence suggests that vitamin D promotes insulin secretion by facilitating calcium influx and upregulating voltage-gated calcium channels in INS-1 cell lines and human islets [14]. Calcitriol (1,25-dihydroxyvitamin D₃), the biologically active metabolite of vitamin D, mediates its physiological effects by binding to vitamin D receptors (VDRs) in target tissues. Substantial evidence indicates that calcitriol plays a protective role in T2DM by lowering disease incidence [15], enhancing pancreatic β-cell function and insulin secretion [16], improving glycemic control [17], ameliorating IR [18], and promoting the healing of diabetic foot ulcers [19]. Furthermore, clinical evidence indicates that calcitriol deficiency has been clinically linked to an increased risk of major T2DM complications, such as hypertension, dyslipidemia, retinopathy, and coronary artery disease [20,21]. Numerous in vivo and ex vivo studies in rats have confirmed that vitamin D deficiency results in lowered serum insulin levels and compromised insulin secretion from isolated islets [22,23]. Conversely, research in mice has indicated that vitamin D supplementation can reverse impaired islet insulin secretion in deficient animals [22–24], supporting a direct regulatory role of vitamin D in islet insulin secretion. Additionally, VDR-mutant mice exhibit significantly reduced serum insulin levels and Ins2 gene expression [24], further suggesting that the vitamin D-vitamin D receptor (VDR) system modulates genes associated with insulin synthesis and secretion. Emerging evidence highlights the dual role of calcitriol in both diabetes and cancer pathophysiology. Recent studies demonstrate its protective effects against β-cell destruction in diabetes mellitus and improvement of insulin signaling in macrophages through ERS inhibition [25,26]. Notably, similar mechanistic interactions have been observed in cancer biology, with current findings revealing a significant association between ERS and calcitriol signaling in both breast cancer and mammary epithelial cell lines [27].However, the specific pharmacological targets and mechanisms underlying the action of calcitriol on T2DM through modulated ERS related pathways remain to be further elucidated.

While prior studies have established the beneficial role of calcitriol in enhancing insulin secretion and protecting pancreatic β-cells against apoptosis, the precise molecular targets and downstream pathways through which it modulates ERS in the context of T2DM remain incompletely understood. Previous *in silico* and *in vitro* studies have frequently concentrated on isolated signaling pathways or narrow sets of targets, thereby lacking a comprehensive view of the underlying mechanistic network. Network pharmacology has emerged as a powerful computational approach in drug discovery, enabling systematic prediction of therapeutic targets and mechanistic exploration of drug actions [11]. In this study, we utilized an integrated network pharmacology approach along with experimental validation in a high glucose-induced MIN6 cell model to systematically identify and validate key molecular targets—STAT3, HSP90AA1, MAPK1, and PIK3R1—that mediate the ameliorative effects of calcitriol on T2DM through modulation of ERS-related pathways. The discovery of this multi-targeted and pathway-integrated mechanism represents a conceptual advance that substantially expands the prevailing—and previously narrow—understanding of calcitriol’s pharmacology, providing fresh mechanistic insight into its therapeutic potential in T2DM.

## Materials and methods

### Screening for calcitriol-associated target genes

**Fig 1:**
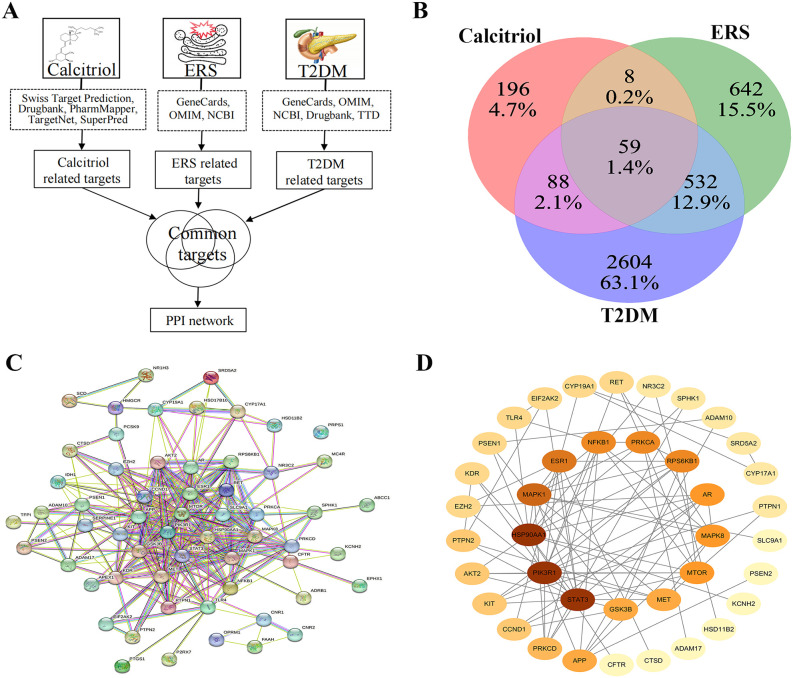
Network pharmacology of calcitriol in the treatment of T2DM. Abbreviations: **(A)** The flowchart of network pharmacology. **(B)** Venn diagram of genes associated with calcitriol, ERS and T2DM. **(C)** PPI network of mutual target genes. **(D)** Potential target genes. Abbreviations: T2DM: type 2 diabetes mellitus; ERS: endoplasmic reticulum stress; PPI: protein–protein interaction.

The network pharmacology research workflow is illustrated in Fig 1A. We obtained the canonical SMILES of calcitriol from PubChem (https://pubchem.ncbi.nlm.nih.gov/) and used them to query multiple databases: Swiss Target Prediction (http://www.swisstargetprediction.ch/) and DrugBank (https://go.drugbank.com/), for calcitriol-associated genes, and PharmMapper (http://www.lilab-ecust.cn/pharmmapper/), TargetNet (http://targetnet.scbdd.com/), and SuperPred (https://bioinformatics.charite.de/superpred/) (with a score threshold ≥ 0.6) for additional gene targets. Furthermore, protein names were converted into their corresponding gene names using the Uniprot database (https://www.uniprot.org/). After deduplication, we compiled a comprehensive set of calcitriol-related target genes. All database queries were performed on February 13, 2023.

### Identification of the target genes associated with ERS and T2DM

We identified ERS-related target genes by searching the GeneCards (https://www.genecards.org/), Online Mendelian Inheritance in Man (OMIM) (https://omim.org/), and National Center for Biotechnology Information (NCBI) (https://www.ncbi.nlm.nih.gov/) databases using the keyword “endoplasmic reticulum stress.” Similarly, T2DM-associated genes were retrieved from GeneCards, OMIM, NCBI, DrugBank, and the Therapeutic Target Database (TTD) (https://db.idrblab.net/ttd/) using the keywords “type 2 diabetes mellitus” and “type 2 diabetes.” All database searches were conducted on February 13, 2023. Only ERS- and T2DM-related genes with a GeneCards score ≥ 30 were retained. Next, we compiled the target genes of calcitriol, ERS, and T2DM and uploaded them to a Venn diagram tool to identify overlapping genes.

### Protein–protein interaction network analysis

The overlapping genes were analyzed using the STRING database (https://cn.string-db.org/) to construct a protein-protein interaction (PPI) network. A high-confidence interaction network (minimum interaction score ≥ 0.9) was generated and then visualized in Cytoscape (v3.7.1). Network topology was assessed using Cytoscape’s built-in “NetworkAnalyzer” and “CytoNCA” plugins, with genes filtered based on three centrality measures: betweenness centrality (BC), closeness centrality (CC), and degree centrality (DC). This analysis yielded three distinct subnetworks. Finally, the top twelve highest-scoring genes from each subnetwork were selected as core targets for subsequent analysis, prioritizing those with mutual interactions.

### Gene ontology and Kyoto encyclopedia of genes and genomes pathways enrichment analysis

To elucidate the functional roles and signaling pathways of the core targets, we conducted integrated bioinformatic analyses using the DAVID database (https://david.ncifcrf.gov/), interrogating Gene Ontology (GO) terms and Kyoto Encyclopedia of Genes and Genomes (KEGG) pathways. The enrichment results were then visualized through an online bioinformatics platform (http://www.bioinformatics.com.cn/).

### Construction of multiple network relationships

The Cytoscape software was utilized to construct an integrated interactive network diagram of drug-target-GO/KEGG pathways-ERS/disease, incorporating the core target genes and their associated GO and KEGG pathways for calcitriol in the treatment of T2DM by modulating ERS.

### Molecular docking

To evaluate the binding interactions between calcitriol and core target proteins, we performed molecular docking simulations. The three-dimensional structure of calcitriol was obtained from the PubChem database (https://pubchem.ncbi.nlm.nih.gov/) and prepared for docking using AutoDock Tools (v1.5.7). The preparation included conversion to mol2 format, addition of hydrogen atoms, definition of torsion trees, and final export in the pdbqt format. For protein preparation, crystal structures of target proteins were acquired from the Protein Data Bank (https://www.rcsb.org/). Each protein structure was processed in AutoDock Vina through hydrogen addition and water molecule removal. The docking grid box was carefully centered to encompass all residues within the original ligand’s binding pocket. PyMOL software was utilized to calculate root-mean-square deviation (RMSD) values of the original ligand molecules for assessing docking parameters setting, with an RMSD value ≤ 4Å generally considered as a threshold for matching ligand molecule configuration. Finally, LigPlot+ (v2.2.5) was employed to analyze and visualize hydrophobic interactions from the docking results.

### Cell culture and treatments

MIN6 cells (Keycell Biotechnology Co., Ltd., Wuhan, China) were cultured in Dulbecco’s Modified Eagle Medium (DMEM; 5.5 mM glucose) containing 10% fetal bovine serum (Global Kang, Qinhuangdao, China), 100 U/mL penicillin, and 100 μg/mL streptomycin at 37°C in a humidified 5% CO₂ atmosphere. For glucose treatment experiments, cells were divided into three experimental groups (n = 3 per group): normal glucose control (NG; 5.5 mM glucose), high glucose treatment (HG; 25 mM glucose), HG+calcitriol treatment group (HG + calcitriol). Glucose concentrations were selected based on established protocols from previous studies [28]. All treatments were performed in biological triplicate.

### Cell viability assay

Cell viability was evaluated using CCK-8 assay in MIN6 cells exposed to HG with or without calcitriol (MedChemExpress, USA). Briefly, cells were seeded in 96-well plates (4 × 10³ cells/well) and pre-cultured in DMEM for 12 h. Subsequently, the medium was replaced with different treatments: NG group (DMEM+5.5 mM glucose), HG group (DMEM+25 mM glucose), and calcitriol groups (DMEM+25 mM glucose+various concentrations of calcitriol). Following 48 hours of treatment incubation, 10 μL of CCK-8 reagent (Keycell Biotechnology, Wuhan, China) was added to each well, followed by a 2-hour incubation at 37°C under light-protected conditions. Absorbance was then measured at 450 nm using a microplate reader (BIOBASE, Jinan, China) to quantify cell viability.

### Insulin and inflammatory cytokines IL-6, TNF-α levels analysis

The effects of calcitriol on the levels of insulin and inflammatory cytokines IL-6 and TNF-α were quantified in HG-pretreated MIN6 cells using the insulin (Elabscience), IL-6 (Proteintech) and TNF-α (Proteintech) Enzyme-Linked Immunosorbent Assay (ELISA) Kits, following the manufacturer’s instructions.

### Cell apoptosis analysis by flow cytometry

Apoptosis rates in MIN6 cells were evaluated using an Annexin V-FITC/PI apoptosis detection kit (Keycell Biotechnology, Wuhan, China). Cells were seeded in 6-well plates at 5 × 10^5^ cells/well and allowed to adhere overnight (37°C, 5% CO_2_). Subsequently, the cells were exposed to HG with or without 10 nM calcitriol for 48 h. Afterwards, cells were harvested and dual-stained with Annexin V-FITC and propidium iodide (PI) for 10 minutes under light-protected conditions. Quantitative apoptosis analysis was performed using a flow cytometer (Beckman Coulter, Brea, CA, USA), with early and late apoptotic populations identified based on Annexin V/PI staining patterns.

### qPCR analysis

The total mRNA was extracted from MIN6 cells following the Trizol manufacturer’s protocols (Ambion). Subsequently, cDNA synthesis was performed using HiScript® II Q Select RT SuperMix (Vazyme, China) and gDNA wiper Mix (Vazyme, China). Real-time reverse transcription polymerase chain reaction (RT-PCR) was conducted on an ABI Prism 7300 real-time thermocycler (Applied Biosystems, Foster City, USA) employing SYBR qPCR Master Mix (ToloBio, China). β-actin served as a housekeeping gene. The relative gene expression levels were normalized using the 2^-ΔΔCT^ method. Primers were designed and synthesized by TsingkeBiotechnologyCo.,Ltd., with their sequences provided in Table 1.

**Table 1:** Primers used for qRT-PCR analysis.

Gene	Primer	Sequence (5’ → 3’)
β-actin	Forward	5’-CACGATGGAGGGGCCGGACTCATC-3’
	Reverse	5’-TAAAGACCTCTATGCCAACACAGT-3’
STAT3	Forward	5’-GTAGAGCCATACACCAAGCAGCAG-3’
	Reverse	5’-AATGTCGGGGTAGAGGTAGACAAGT-3’
HSP90AA1	Forward	5’-CCCTGACCATTGTGGATACC-3’
	Reverse	5’-GACTCCCAGGCATACTGCTC-3’
MAPK1	Forward	5’-TCCTTTTGAGCACCAGACCT-3’
	Reverse	5’-AAAGGTCCGTCTCCATGAGG-3’
PIK3R1	Forward	5’-AATGCACGGCGATTACACTC-3’
	Reverse	5’-GGACACTGGGTAGAGCAACT-3’

### Western blot analysis

Total protein was extracted from MIN6 cells using RIPA lysis buffer (Servicebio, Wuhan, China) supplemented with protease inhibitors. Protein concentrations were quantified using a BCA assay kit (GBCBIO Technologies, Guangzhou, China). Equal amounts of protein (40 μg per lane) were resolved by 10% SDS-PAGE and electrotransferred to polyvinylidene difluoride membranes (Millipore, Billerica, MA, USA). After blocking with 5% non-fat milk in TBST for 2 h at room temperature, membranes were incubated overnight at 4°C with the following primary antibodies: rabbit monoclonal anti-STAT3 (1:1000, Beyotime), rabbit polyclonal anti-HSP90AA1 (1:2000, Proteintech), rabbit polyclonal anti-MAPK1 (1:1000, Invitrogen), rabbit polyclonal anti-PIK3R1 (1:1000, Immunoway), and mouse monoclonal anti-β-actin antibody (1:5000,Affinity). Subsequently, the membranes were washed five times with Tris-buffered saline containing Tween 20 for 5 minutes each time and then incubated with HRP-conjugated secondary antibody(1:10000; Proteintech) at room temperature for 120 min. Protein bands were detected using an ECL Plus kit (Servicebio) and quantified by densitometry using ImageJ software, with all protein levels normalized to β-actin expression.

## Results

### Identification of calcitriol, ERS and T2DM Related Genes

The molecular characteristics of calcitriol were obtained from PubChem database: 5280453 for the CID, C_27_H_44_O_3_ for the chemical formula, 416.6 g/mol for the molecular weight, and [CC(CCCC(C)(C)O)C1CCC2C1(CCCC2 = CC = C3CC(CC(C3 = C)O)O)C] for the canonical SMILES. A total of 351 genes related to calcitriol were identified through a search on specified databases and subsequent elimination of duplicates. Additionally, by removing duplicates separately, we obtained a total of 1241 genes related to ERS and 3283 genes related to T2DM (S1 Table). Venn diagram analysis revealed 59 overlapping targets among these three gene sets (Fig 1B). These common targets were subsequently used to construct an interlaced network (Fig 1C).

### Network Analysis and Core Targets

**Fig 2:**
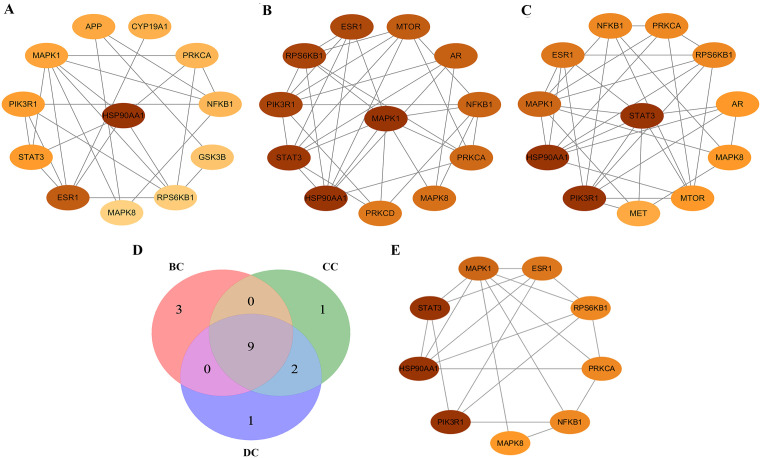
Topological analyses of the potential target genes. **(A)** Top twelve potential target genes were identified based on their BC. **(B)** Top twelve potential target genes were identified based on their CC. **(C)** Top twelve potential target genes were identified based on their DC. **(D)** Venn diagram of the first twelve BC, CC and DC target genes. **(D)** Core target genes. Abbreviations: BC: betweenness centrality; CC: closeness centrality; DC: degree centrality.

The intersected target genes were analyzed using a high-confidence PPI network (interaction score ≥ 0.9), constructed and visualized in Cytoscape (Fig 1D). Topological parameters including BC, CC, and DC were computed using the CytoNCA plugin to identify hub genes. From these analyses, we selected the top twelve highest-scoring genes for each centrality measure (BC, CC, and DC). Genes common to all three centrality clusters were defined as core targets: STAT3, HSP90AA1, MAPK1, PIK3R1, ESR1, RPS6KB1, NFKB1, PRKCA, and MAPK8 (Fig 2; S2 Table).

### Enrichment Analysis

**Fig 3:**
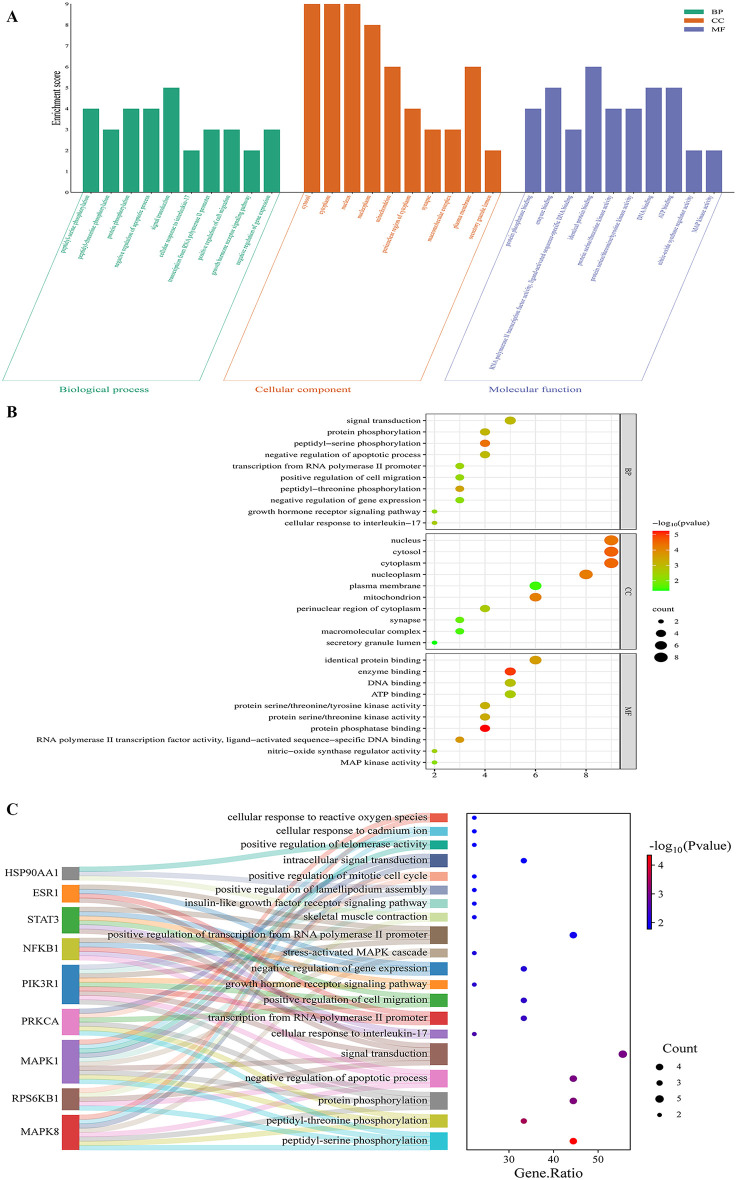
GO enrichment analyses of the core target genes for calcitriol in the treatment of T2DM by modulating ERS. **(A)** Bar chart, **(B)** Bubble chart, **(C)** Sankey map. Abbreviations: GO: Gene Ontology; T2DM: type 2 diabetes mellitus; ERS: endoplasmic reticulum stress.

**Fig 4:**
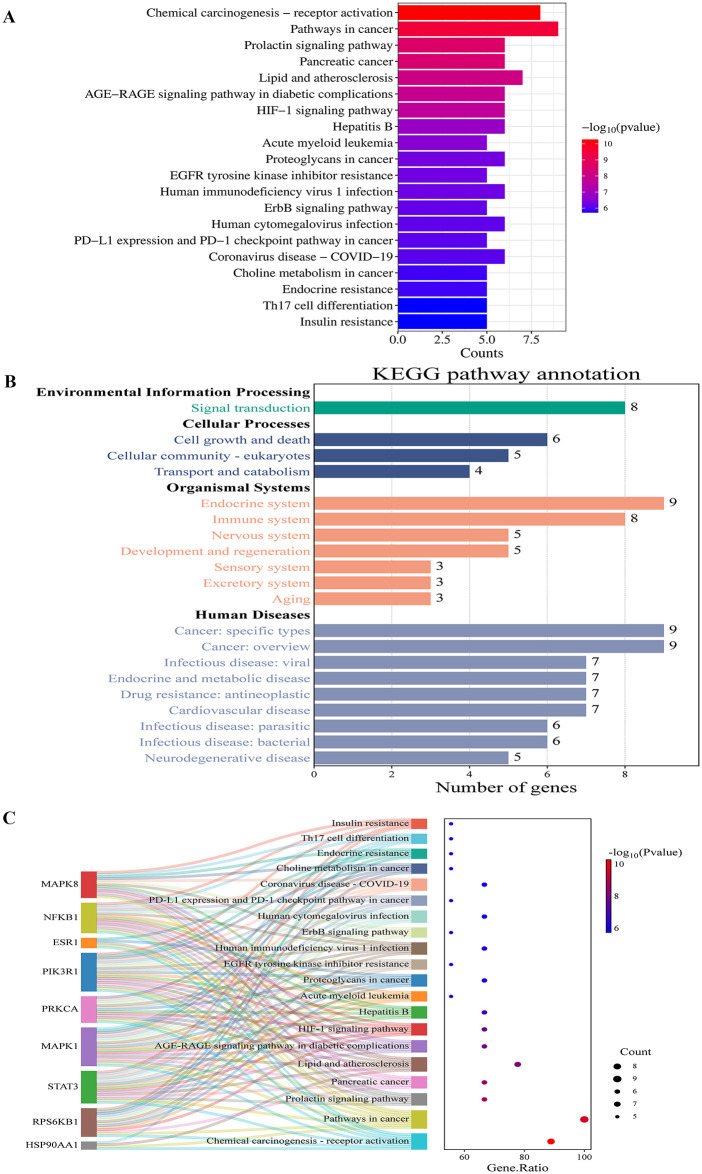
KEGG enrichment analyses of the core target genes for calcitriol in the treatment of T2DM by modulating ERS. **(A)** Bar chart, **(B)** Classified chart, **(C)** Sankey map. Abbreviations: KEGG: Kyoto Encyclopedia of Genes and Genomes; T2DM: type 2 diabetes mellitus; ERS: endoplasmic reticulum stress.

GO enrichment analysis for biological processes (BP) demonstrated that the core targets were predominantly involved in peptidyl-serine phosphorylation, peptidyl-threonine phosphorylation, protein phosphorylation, negative regulation of apoptotic process, signal transduction, etc. In addition, cellular components (CC) enrichment analysis localized the core targets to major subcellular compartments. High enrichment was observed in cytosol, cytoplasm, nucleus, nucleoplasm, mitochondrion etc. Furthermore, molecular functions (MFs) attributed to the core targets were predominantly involved in protein phosphatase binding, enzyme binding, RNA polymerase II transcription factor activity, ligand-activated sequence-specific DNA binding, identical protein binding, protein serine/threonine kinase activity, etc. These findings were depicted in the bar chart (Fig 3A, S3-S5 Tables), the bubble chart (Fig 3B), and the Sankey map (Fig 3C). Furthermore, KEGG enrichment analysis revealed the identification of 101 KEGG pathways associated with the core target genes (p ＜ 0.05). These pathways include lipid and atherosclerosis, AGE-RAGE signaling pathway in diabetic complications, HIF-1 signaling pathway, EGFR tyrosine kinase inhibitor resistance, ErbB signaling pathway, endocrine resistance, Th17 cell differentiation, and insulin resistance (Fig 4; S6-S7 Tables).

### Multiple Network Relationships

**Fig 5:**
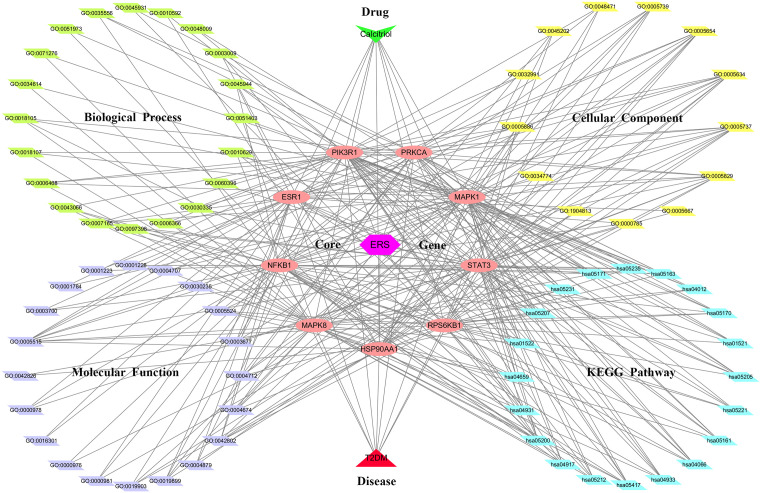
A multiple merged network of drug–targets–GO terms and KEGG pathways–ERS/T2DM. Abbreviations: GO: Gene Ontology; KEGG: Kyoto Encyclopedia of Genes and Genomes; T2DM: type 2 diabetes mellitus; ERS: endoplasmic reticulum stress.

A comprehensive network integration was performed, encompassing drug-target interactions, GO terms (BP, CC, MF), KEGG pathways, and associations with diseases and endoplasmic reticulum stress (ERS). This analysis revealed that the therapeutic effect of calcitriol on T2DM involves targeting ERS across multiple signaling pathways (Fig 5).

### Molecular docking validation

For molecular docking validation, we selected proteins corresponding to the core target genes STAT3, HSP90AA1, MAPK1, and PIK3R1. These were prioritized based on their highest composite scores integrating BC, CC, and DC metrics, and were further supported by established literature linking them to calcitriol signaling and β‑cell function. [29–36]. In this study, the structures of STAT3 (PDB ID: 6NJS), HSP90AA1 (PDB ID: 7LT0), MAPK1 (PDB ID: 7NR9), and PIK3R1 (PDB ID: 4JPS) were used to model their interactions with calcitriol through AutoDock simulations. The grid box parameters and corresponding docking results for all four targets are presented in Table 2.The grid box settings for STAT3 were defined as follows: 13.498, 54.117, and 0.100 for the central coordinates in the x-y-z plane, and 15, 21, and 15 for the dimensions along the x-y-z axes. In the 6NJS protein binding site, the original ligand KQV was observed to be docking with the MET-660 (3.4 Å), LYS-658 (2.3 Å), TYR-657 (2.7 Å, 3.3 Å), SER-636 (2.0 Å), ARG-609 (2.8 Å), SER-613 (2.7 Å, 2.9 Å), SER-611 (2.7 Å, 3.2 Å) and GLU-612 (3.2 Å) amino acid residues of 6NJS with eleven hydrogen bonds, and the affinity and RMSD were −9.5 kcal/mol and1.646 Å, respectively (S1A Fig). Calcitriol was found to be bonding with 6NJS through the GLN-644 (2.9 Å), TYR-640 (2.3 Å), GLN-635 (3.2 Å) and GLU-625 (2.3 Å) amino acid residues with an affinity of −6.5 kcal/mol (S1B Fig). Additionally, the grid box settings for HSP90AA1 were defined as follows: −31.316, −10.22, and −24.379 for the central coordinates in the x-y-z plane, and 15, 15, and 15 for the dimensions along the x-y-z axes. In the 7LT0 protein binding site, the original ligand ONJ was observed to be docking with the THR-184 (2.7 Å) amino acid residue of 7LT0 with a hydrogen bond, and the affinity and RMSD were −11.5 kcal/mol and 0.194 Å, respectively (S1C Fig). Calcitriol was found to be bonding with 7LT0 through the GLY-97 (2.0 Å) amino acid residue with an affinity of −8.4 kcal/mol (S1D Fig). Furthermore, the grid box settings for MAPK1 were defined as follows: 5.915, 8.737, and 45.325 for the central coordinates in the x-y-z plane, and 17.25, 15, and 15 for the dimensions along the x-y-z axes. In the 7NR9 protein binding site, the original ligand UOW was observed to be docking with the THR-68 (2.9 Å), ASP-167 (2.6 Å), TYR-36 (2.8 Å), GLN-105 (2.1 Å), LYS-54 (3.5 Å) and MET-108 (1.9 Å, 3.1 Å) amino acid residues of 7LT0 with seven hydrogen bonds, and the affinity and RMSD were −10.3 kcal/mol and 3.564 Å, respectively (S1E Fig). Calcitriol was found to be bonding with 7NR9 through the MET-108 (2.1 Å) amino acid residue with an affinity of −9.5 kcal/mol (S1F Fig). Moreover, the grid box settings for PIK3R1 were defined as follows: −1.166, −8.926, and 16.981 for the central coordinates in the x-y-z plane, and 15, 15, and 15 for the dimensions along the x-y-z axes. In the 4JPS protein binding site, the original ligand 1LT was observed to be docking with the GLN-859 (1.9 Å, 2.9 Å), SER-854 (1.9 Å) and VAL-851 (2.1 Å, 2.5 Å) amino acid residues of 4JPS with five hydrogen bonds, and the affinity and RMSD were −10.3 kcal/mol and 0.095 Å, respectively (S1G Fig). Calcitriol was found to be bonding with 4JPS through the LYS-802 (3.0Å) and ARG-770 (3.2Å) amino acid residues with an affinity of −7.6 kcal/mol (S1H Fig).

**Table 2:** The grid box parameters and docking results for the ligands and the four targets.

Receptor	PBD ID	Grid box	Ligand	Affinity (kcal/mol)	Hydrogen bond	Hydrophobic bond
STAT3	6NJS	Center: x = 13.498 y = 54.117 z = 0.100 Size: x = 13.498 y = 54.117 z = 0.100	KQV	−9.5	MET-660 (3.4 Å), LYS-58 (2.3 Å), TYR-657 (2.7 Å, 3.3 Å), SER-636 (2.0 Å), ARG-609 (2.8 Å), SER-613 (2.7 Å, 2.9 Å), SER-611 (2.7 Å, 3.2 Å), GLU-612 (3.2 Å)	Ile659, Gln635, Trp623, Pro639, Tyr640, Glu638, Gln644, Val637
			Calcitriol	−6.5	GLN-644 (2.9 Å), TYR-640 (2.3 Å), GLN-635 (3.2 Å), GLU-625 (2.3 Å)	Trp623, Ile659, Ser636, Glu638, Val637, Gln635, Tyr657
HSP90AA1	7LT0	Center: x = −31.316 y = −10.220 z = −24.379 Size: x = 13.498 y = 54.117 z = 0.100	ONJ	−11.5	THR-184 (2.7 Å)	Val150, Val186, Asp93, Leu48, Ser52, Met98, Gly97, Ile96, Lys58, Phe138, Trp162, Leu103, Asn51, Ala55, Asp54
			Calcitriol	−8.4	GLY-97 (2.0 Å)	Leu103, Trp162, Leu107, Asp102, Lys58, Ile96, Phe138, Asn51, Ala55, Asp54
MAPK1	7NR9	Center: x = 5.915 y = 8.737 z = 45.325 Size: x = 13.498 y = 54.117 z = 0.100	UOW	−10.3	THR-68 (2.9 Å), ASP-167 (2.6 Å), TYR-36 (2.8 Å), GLN-105 (2.1 Å), LYS-54 (3.5 Å), MET-108 (1.9 Å, 3.1 Å)	Ile56, Glu71, Arg67, Asp167, Asp111, Val39, Ile31, Cys166, Lys54, Leu156, Ile84, Tyr36, Asn154, Ser153, Ala52, Leu107, Asp106
			Calcitriol	−9.5	MET-108 (2.1 Å)	Arg67, Glu71, Tyr36, Cys166, Ser153, Val39, Leu156, Thr68, Ile56, Asp167, Lys54, Ala52, Ile31, Thr110, Glu109
PIK3R1	4JPS	Center: x = −1.166 y = −8.926 z = 16.981 Size: x = 13.498 y = 54.117 z = 0.100	1LT	−10.3	GLN-859 (1.9 Å, 2.9 Å), SER-854 (1.9 Å), VAL-851 (2.1 Å, 2.5 Å)	Val850, Phe930, Ile932, Tyr836, Ile848, Met922, Ile800, His855, Trp780
			Calcitriol	−7.6	LYS-802 (3.0 Å), ARG-770 (3.2 Å)	Trp780, Val851, Val850, Met922, Tyr836, Ile848, Ile932, Asp933, Gln859, Ser854, Ile800

In addition, we uploaded the docking results of the ligands and the core target proteins to the LigPlot+ software to further identify their hydrophobic interactions (Table 2). The original ligand KQV was found to be interacting with the Ile659, Gln635, Trp623, Pro639, Tyr640, Glu638, Gln644 and Val637 amino acid residues of the 6NJS protein with eight hydrophobic bonds (S2A Fig). The hydrophobic bonding between calcitriol and the 6NJS protein shows an interaction with seven bonds, involving the Trp623, Ile659, Ser636, Glu638, Val637, Gln635 and Tyr657 amino acid residues (S2B Fig). The original ligand ONJ was found to be interacting with the Val150, Val186, Asp93, Leu48, Ser52, Met98, Gly97, Ile96, Lys58, Phe138, Trp162, Leu103, Asn51, Ala55 and Asp54 amino acid residues of the 7LT0 protein with fifteen hydrophobic bonds (S2C Fig). The hydrophobic bonding between calcitriol and the 7LT0 protein shows an interaction with ten bonds, involving the Leu103, Trp162, Leu107, Asp102, Lys58, Ile96, Phe138, Asn51, Ala55 and Asp54 amino acid residues (S2D Fig). The original ligand UOW was found to be interacting with the Ile56, Glu71, Arg67, Asp167, Asp111, Val39, Ile31, Cys166, Lys54, Leu156, Ile84, Tyr36, Asn154, Ser153, Ala52, Leu107, and Asp106 amino acid residues of the 7NR9 protein with seventeen hydrophobic bonds (S2E Fig). The hydrophobic bonding between calcitriol and the 7NR9 protein shows an interaction with fifteen bonds, involving the Arg67, Glu71, Tyr36, Cys166, Ser153, Val39, Leu156, Thr68, Ile56, Asp167, Lys54, Ala52, Ile31, Thr110 and Glu109 amino acid residues (S2F Fig). The original ligand 1LT was found to be interacting with the Val850, Phe930, Ile932, Tyr836, Ile848, Met922, Ile800, His855 and Trp780 amino acid residues of the 4JPS protein with nine hydrophobic bonds (S2G Fig). The hydrophobic bonding between calcitriol and the 4JPS protein shows an interaction with eleven bonds, involving the Trp780, Val851, Val850, Met922, Tyr836, Ile848, Ile932, Asp933, Gln859, Ser854, Ile800 amino acid residue (S2H Fig). These results revealed that calcitriol and the original ligands share a common set of key hydrophobic residues within the binding pockets, suggesting a similar binding mode. This similarity in binding mode likely contributes to their comparable binding affinities, although minor differences in polar interactions or desolvation penalties cannot be excluded.

### Effect of calcitriol on MIN6 cell viability

**Fig 6:**
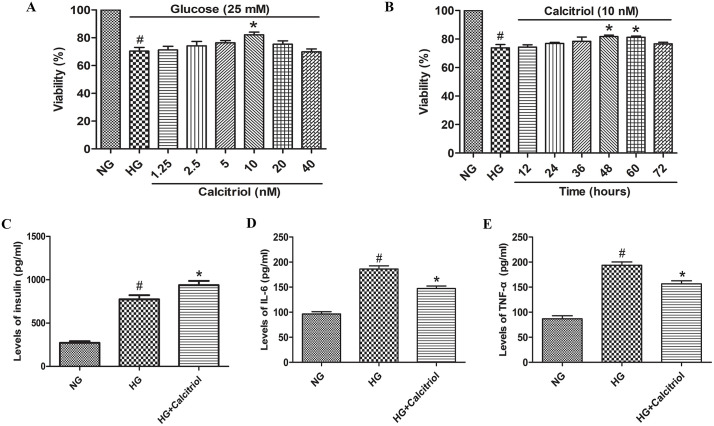
Effect of calcitriol on the viability and inflammatory cytokines levels in HG-induced MIN6 cells. **(A)** The cell viability of HG-induced MIN6 cells with various concentrations of calcitriol pretreatment for 48 h was detected by a Cell Counting Kit-8 assay. **(B)** The cell viability of HG-induced MIN6 cells with 10 nM calcitriol pretreatment for different time periods was measured. **(C)** Calcitriol inhibits the levels of IL-6 in HG-induced MIN6 cells. **(D)** Calcitriol inhibits the levels of TNF-α in HG-induced MIN6 cells. Data are shown as the mean  ±  SD (n  =  3). ^#^*p* < 0.05 compared with NG group; ^*^p < 0.05 compared with HG group. Abbreviations: HG: high glucose.

First, we evaluated the impact of calcitriol on MIN6 cell viability across a range of concentrations during a 48-hour treatment period. Initial experiments demonstrated that exposure to HG conditions (25 mmol/L for 48 hours) significantly reduced MIN6 cell viability. However, calcitriol treatment dose-dependently restored cell viability, with maximal protective effects observed at 10 nM concentration (Fig 6A). Time-course experiments further revealed that 10 nM calcitriol significantly enhanced cell viability after both 48 and 60 hours of treatment compared to high glucose controls (Fig 6B). Based on these results, we selected 10 nM calcitriol with a 48-hour treatment duration for subsequent investigations of its protective effects against high glucose-induced cytotoxicity.

### Effect of calcitriol on the levels of insulin, IL-6 and TNF-α in HG-induced MIN6 cells

As depicted in Fig 6C-D, the levels of insulin, IL-6 and TNF-α were found to be upregulated in the HG group compared to the NG group. However, pretreatment with calcitriol obviously increased insulin secretion and normalized the elevated concentrations of inflammatory cytokines including IL-6 and TNF-α.

### Calcitriol prevented MIN6 cells from HG-induced apoptosis

**Fig 7:**
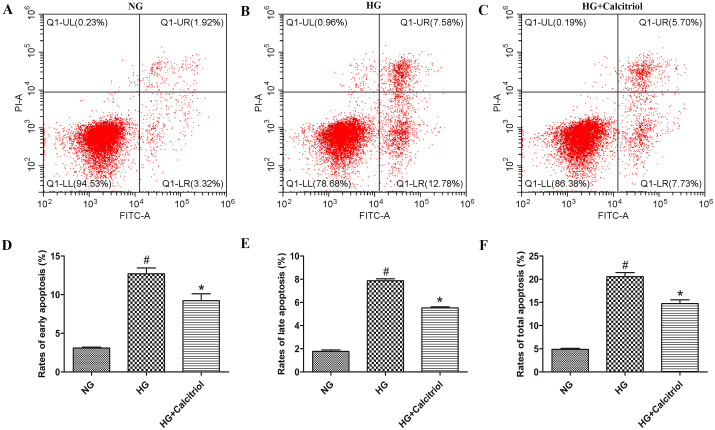
Calcitriol prevented MIN6 cells from HG-induced apoptosis using an AnnexinV-FITC/PI flow cytometry analysis. **(A)** The representative image of NG group. **(B)** The representative image of HG group. **(C)** The representative image of calcitriol group. **(D)** The rates of early apoptotic MIN6 cells in NG, HG and calcitriol groups. **(E)** The rates of late apoptotic MIN6 cells in NG, HG and calcitriol groups. **(F)** The rates of total apoptotic MIN6 cells in NG, HG and calcitriol groups. Data are shown as the mean  ±  SD (n  =  3). ^#^*p* < 0.05 compared with NG group; ^*^p < 0.05 compared with HG group. Abbreviations: HG: high glucose; NG: normal group.

MIN6 cell apoptosis was quantified by flow cytometry using a dual-labeling approach (Annexin V-FITC and PI), which distinguishes early apoptotic (Annexin V + /PI−) from late apoptotic/necrotic (Annexin V + /PI+) populations. HG treatment for 48 hours significantly increased apoptosis, with early apoptosis rising to 12.69%, late apoptosis to 7.86%, and total apoptosis reaching 20.55%. Notably, calcitriol treatment reduced these effects, lowering early apoptosis to 9.21%, late apoptosis to 5.52%, and total apoptosis to 14.73% (Fig 7 and S1 file). These results indicate that HG-induced MIN6 cell death occurs predominantly via apoptosis rather than necrosis.

### Effect of calcitriol on the mRNA/protein expression of core targets in HG-induced MIN6 cells

**Fig 8:**
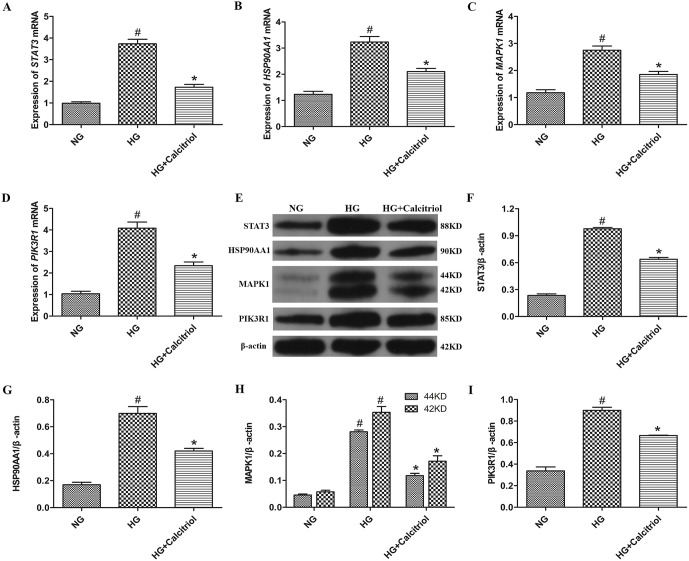
Effect of calcitriol on the mRNA/protein expression of core targets in HG-induced MIN6 cells. (A) mRNA expression of STAT3 in MIN6 cells. (B) mRNA expression of HSP90AA1 in MIN6 cells. (C) mRNA expression of MAPK1 in MIN6 cells. (D) mRNA expression of PIK3R1 in MIN6 cells. **(E)** STAT3, HSP90AA1, MAPK1 and PIK3R1 protein expression levels were measured by Western blot analysis. **(F)** Quantification of STAT3 protein expression. **(G)** Quantification of HSP90AA1 protein expression. **(H)** Quantification of MAPK1 protein expression. **(I)** Quantification of PIK3R1 protein expression. Data are shown as the mean  ±  SD (n  =  3). ^#^*p* < 0.05 compared with NG group; ^*^p < 0.05 compared with HG group. Abbreviations: NG: normal group; HG: high glucose.

Following network pharmacology analysis, which identified STAT3, HSP90AA1, MAPK1, and PIK3R1 as key targets, we developed a HG-induced MIN6 cell model to assess the regulatory effects of calcitriol on their mRNA and protein expression. As illustrated in Fig 8 and S2 File, HG exposure significantly upregulated the mRNA levels of these targets compared to the NG group, whereas calcitriol treatment markedly suppressed their expression. Similarly, protein expression of STAT3, HSP90AA1, MAPK1, and PIK3R1 was substantially increased under HG conditions but was effectively attenuated by calcitriol.

## Discussion

T2DM has emerged as a major global health challenge, largely driven by the rising pandemic of obesity and its association with severe chronic complications, including increased risks of diabetic nephropathy, cardiovascular disorders, non-alcoholic hepatic steatosis, and diabetic retinopathy [37]. The development of chronic complications in T2DM patients represents a major driver of the disease’s substantial economic burden, comprising approximately 67% of total diabetes-related healthcare expenditures. While novel hypoglycemic agents have demonstrated beneficial effects on diabetic nephropathy and cardiovascular outcomes, the global prevalence of T2DM and its associated complications continues to rise at an alarming rate [38]. This study employed an integrated approach combining network pharmacology and molecular docking to systematically identify potential target genes, biological functions, and signaling pathways through which calcitriol may ameliorate T2DM via ERS modulation. Our findings demonstrated that calcitriol’s therapeutic effects on T2DM are mediated through regulation of ERS-related pathways by targeting a specific cluster of key molecules. Experimental validation using HG-induced MIN6 cells further confirmed the predicted mechanisms of calcitriol action.

VDRs are widely expressed in pancreatic tissue, where their signaling is centrally involved in promoting cellular survival, suppressing apoptotic pathways, and maintaining pancreatic β-cell health [39,40]. Calcitriol acts directly on pancreatic β‑cells by binding to the VDR expressed in these cells, subsequently initiating the transcription of genes involved in glucose transport, insulin secretion, and β‑cell proliferation [41]. More broadly, calcitriol modulates inflammatory, oxidative stress responses, ERS, and insulin signaling pathways, underscoring its pleiotropic role in metabolic homeostasis [19]. In our study, we found that calcitriol mediates its therapeutic effects against type 2 diabetes through a multi-mechanistic action, modulating the expression of genes central to disease pathology, including STAT3, HSP90AA1, MAPK1, and PIK3R1. As a member of the STAT protein family, STAT3 has been extensively implicated in the pathogenesis of T2DM. The involvement of STAT3 in cellular differentiation has been extensively demonstrated across diverse cell types, including those within the immune, nervous, and endocrine systems, as well as its role in maintaining pluripotent stem cells [42,43]. STAT3 has been demonstrated to be indispensable for the development and functionality of pancreatic β-cells [44], playing a pivotal role in the negative regulation of the PTEN-AKT signaling pathway associated with pancreatic β-cell dysfunction and apoptosis [30]. Pharmacological inhibition of STAT3 signaling markedly enhanced β-cell reprogramming efficiency in both in vitro and in vivo models, facilitating islet-like cluster formation in pancreatic tissue and significantly improving glycemic control in diabetic mice [45]. Furthermore, STAT3 plays a crucial role in hepatic insulin resistance through IL-6-dependent mechanisms, where IL-6/STAT3 axis activation leads to suppression of hepatic gluconeogenic pathways [46]. Moreover, STAT3 has a dual role in the ERS response in β-cells. Acute ERS transiently activates STAT3 to support β-cell survival [47]; however, under conditions of chronic or unresolved ERS, sustained STAT3 signaling may become maladaptive. This persistent activation can promote β-cell dysfunction by modulating the expression of key ERS effectors, ultimately shifting the cellular fate from adaptive homeostasis toward apoptotic demise [48]. HSP90α, encoded by the HSP90AA1 gene, is a member of the heat shock protein family that functions as a molecular chaperone. Emerging evidence indicates that cytokine-induced ERS triggers HSP90α release via activation of the JNK signaling pathway, subsequently promoting pancreatic β-cell apoptosis [32]. Importantly, HSP90AA1 expression dynamics appear to critically regulate islet adaptation to hyperglycemic conditions and may contribute to early atherosclerotic inflammatory injury in T2DM [49, 50]. Furthermore, HSP90AA1 serves as a critical molecular chaperone with an indispensable role in the ERS response. It is fundamentally required to manage the increased burden of client protein folding that characterizes the onset of ERS [51]. Conversely, the sustained extracellular release of HSP90AA1 can paradoxically activate pro-apoptotic signaling cascades. This functional duality positions HSP90AA1 at the nexus of the unfolded protein response (UPR) and apoptotic pathways, acting as a key determinant of cell fate under ERS [32]. MAPK1 (ERK2), a central component of the MAP kinase family, plays critical roles in regulating cellular apoptosis, proliferation, and differentiation [52]. Substantial evidence links ERK signaling to both insulin action and T2DM pathogenesis [34]. Notably, MAPK1 hyperactivation induces inhibitory serine phosphorylation at multiple IRS-1 sites (Ser307/612/632/636), thereby suppressing tyrosine phosphorylation and impairing IRS-1 function [53]. This disruption of insulin signaling promotes insulin resistance development [54]. While physiological insulin stimulation activates ERK signaling to mediate normal cellular proliferation and differentiation [55]. Pathological conditions lead to ERK overactivation through multiple stimuli including growth factors, cytokines, and viral infections. Such excessive ERK activation cross-talks with and impairs insulin signaling transduction [52]. Critically, MAPK1 is a well-characterized downstream component of ERS signal transduction, functioning primarily as an effector of the IRE1 endoribonuclease within the UPR [56]. Engagement of the IRE1-MAPK1 axis by unresolved ERS directly contributes to β-cell apoptosis and, via the serine phosphorylation of IRS-1, promotes a feed-forward loop that exacerbates IR [57]. PIK3R1 encodes p85α, the regulatory subunit of phosphoinositide 3-kinase (PI3K), which associates with the p110 catalytic subunit to form the functional PI3K heterodimer. As a central node in insulin signaling, PI3K regulates multiple cellular processes including glucose uptake, metabolism, cell survival, proliferation, migration, and glycogen synthesis [36]. Through mTOR inhibition, the PI3K signaling pathway activates autophagy by modulating downstream components of the autophagy cascade, ultimately promoting autolysosome formation [58]. This autophagic process enables clearance of misfolded proteins and damaged organelles while attenuating inflammatory responses and preserving cellular homeostasis [59]. Notably, genetic studies by Terauchi et al. revealed that PIK3R1 knockout mice exhibited improved glucose tolerance, enhanced insulin sensitivity, and spontaneous hypoglycemia, underscoring the critical involvement of p85α in insulin resistance pathogenesis [60]. In addition to its canonical function in insulin signaling, PIK3R1 delineates a direct mechanistic link to the UPR. The p85α subunit has been demonstrated to engage in a stress-dependent interaction with XBP-1, a master regulator of the UPR. This association is critical for facilitating the nuclear accumulation of XBP-1, thereby initiating an adaptive transcriptional program that promotes β-cell survival under ERS [61]. Furthermore, The significance of PIK3R1 in this pathway is further supported by bioinformatics data identifying it as a hub gene associated with ERS in pancreatic diseases [62].

KEGG pathway analysis revealed that the four core targets (STAT3, HSP90AA1, MAPK1, PIK3R1) converge on and critically modulate several key signaling pathways implicated in T2DM pathogenesis, namely HIF-1, PI3K-Akt, TNF, FoxO, and insulin signaling pathways. These targets form an interactive network within these pathways to potentially mediate the effects of calcitriol. Specifically, STAT3 and MAPK1 (ERK2) are pivotal upstream regulators and effectors within the HIF-1 and TNF signaling pathways. Their activation can induce HIF-1α expression and TNF-α production, both of which are central to inflammation and insulin resistance [63,64]. The PI3K-Akt pathway, directly targeted by PIK3R1 (the regulatory subunit p85α of PI3K), serves as a central hub [65]. HSP90AA1 stabilizes multiple client proteins including PI3K and Akt, thereby maintaining the integrity of this pathway [65,66]. The activation of the PI3K-Akt cascade, potentially influenced by calcitriol through these core targets, counteracts the negative effects of TNF signaling and promotes insulin sensitivity [64–66]. Conversely, TNF-α signaling, reinforced by STAT3/MAPK1, can inhibit insulin signaling and PI3K-Akt activation via serine phosphorylation of insulin receptor substrates, a process that may be mitigated by the modulation of these core targets [64,67]. Furthermore, the FoxO signaling pathway, which is negatively regulated by Akt phosphorylation, integrates signals from the PI3K-Akt and inflammatory (TNF) pathways to fine-tune gluconeogenesis and β-cell function [65,68]. In summary, STAT3 and MAPK1 primarily mediate pro-inflammatory and stress responses, PIK3R1 and the HSP90AA1-stabilized PI3K-Akt axis are central to metabolic signal transduction, and their cross-talk determines the balance between insulin sensitivity and resistance. Calcitriol may exert its beneficial effects in T2DM by coordinately modulating this multi-target network, thereby downregulating inflammatory (HIF-1, TNF) and upregulating metabolic (PI3K-Akt, insulin) signaling, with FoxO serving as an integrative node.

**Fig 9:**
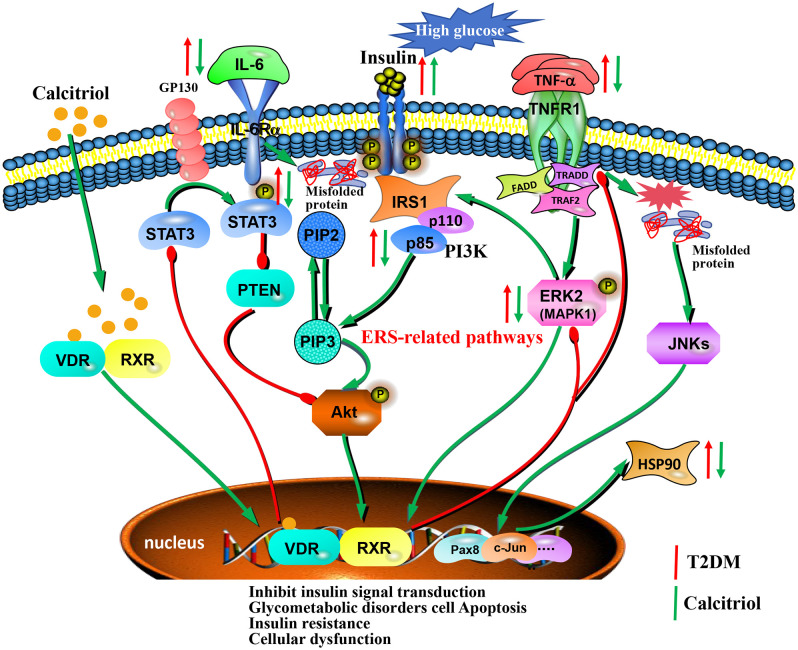
Potential multi-target mechanisms of calcitriol against T2DM. Abbreviations: T2DM: type 2 diabetes mellitus; ERS: endoplasmic reticulum stress.

Cumulative evidence from these studies establishes that the core targets STAT3, HSP90AA1, MAPK1, and PIK3R1 are critically involved in pancreatic β-cell insulin resistance and anti-apoptotic processes, playing fundamental roles in T2DM pathogenesis. In our investigation, we employed integrated molecular docking and molecular biology approaches to analyze both the binding affinities and expression patterns of these key targets. Molecular docking, as a powerful computational tool, has become indispensable for elucidating ligand-receptor interactions, facilitating drug discovery, and informing structural optimization of therapeutic compounds [69]. Molecular docking analysis indicated stable binding of calcitriol to all four candidate targets, primarily mediated by hydrogen bonds and hydrophobic interactions. Notably, the calculated binding affinities for STAT3 and PIK3R1 were lower than those for the other targets, suggesting possible differences in interaction stability. This affinity difference implies that the initial binding of calcitriol to STAT3 and PIK3R1 may be less stable under the simulated conditions. However, it should be noted that a moderately weaker predicted affinity does not preclude biologically relevant interactions and may reflect a regulatory rather than a high-affinity inhibitory role. Subsequent experimental validation confirmed the biological relevance of these interactions. In addition, our findings revealed that calcitriol exerts dual protective effects on MIN6 cells by preventing HG-induced apoptosis while stimulating insulin secretion. In contrast, it was also reported that calcitriol reduces insulin content and secretion as well as increases apoptosis in insulinoma cells, solid β-cell tumors and isolated islets [70]. These divergent effects may be attributed to multiple factors including β-cell heterogeneity, calcitriol concentration, and metabolic conditions. Furthermore, qRT-PCR and western blot analysis demonstrated that exposure to HG significantly upregulated the mRNA and protein expression levels of STAT3, HSP90AA1, MAPK1, and PIK3R1 in MIN6 cells compared to the NG group. Notably, treatment with calcitriol markedly reduced the expression of these targets, suggesting their potential involvement in calcitriol’s anti-T2DM effects. Integrating evidence from the literature and KEGG pathway analysis with our experimental findings, we propose that calcitriol modulates type 2 diabetes mellitus (T2DM) through a multi-targeted mechanism, engaging multiple signaling pathways (Fig 9).

Beyond the mechanistic insights derived from molecular and pathway analyses, our findings may hold considerable translational potential for clinical applications. The identification of specific targets—STAT3, HSP90AA1, MAPK1, and PIK3R1—through which calcitriol ameliorates ERS and enhances β-cell function provides a rational mechanistic basis for its therapeutic use in T2DM, particularly among vitamin D-deficient patients. This subpopulation, which frequently exhibits suboptimal glycemic control and heightened risks of diabetic complications [17,21], may derive significant clinical benefit from targeted vitamin D supplementation or calcitriol-based treatment strategies [71]. Moreover, calcitriol’s dual capacity to inhibit apoptosis and stimulate insulin secretion underscores its potential relevance for personalized medicine paradigms, in which patient stratification based on vitamin D status and target expression profiles could enhance therapeutic precision. Future clinical studies should focus on validating these mechanisms in human trials and identifying predictive biomarkers of treatment response to calcitriol interventions in diabetic populations.

## Conclusion

In conclusion, our study, combining network pharmacology and *in vitro* validation, elucidates the potential core targets and underlying mechanisms through which calcitriol ameliorates T2DM by modulating ERS-related pathways. We demonstrated that calcitriol significantly downregulates four key targets—STAT3, HSP90AA1, MAPK1, and PIK3R1—thereby exerting its anti-T2DM effects. These findings provide novel insights into the therapeutic potential of calcitriol and highlight its regulatory role in T2DM treatment. Nevertheless, *in vivo* and clinical validation in further studies is needed to support these findings.

## Limitations of the study

The current study has several limitations that should be considered when interpreting the results. Firstly, the databases used for predicting potential targets lack comprehensiveness and are not updated in a timely manner. As research on calcitriol progresses, more accurate network pharmacology predictions are expected to become available. Secondly, the core targets of calcitriol were only validated in vitro using HG-induced MIN6 cells. It is important to note that MIN6 is a murine β-cell line, and findings from this model may not fully translate to human islets due to species-specific differences. Furthermore, the concentration of calcitriol applied (10 nM) may not reflect physiologically relevant or therapeutically achievable levels in human systems. Additionally, the network pharmacology predictions are inherently constrained by the accuracy and coverage of the underlying databases, which can vary and influence outcome reliability. Finally, a key limitation of this study is the lack of direct assessment of ERS markers, despite its focus on ERS-related pathways. This gap calls for future investigations to directly visualize ER ultrastructure and quantify key regulatory factors to provide more definitive mechanistic evidence. Therefore, future studies involving in vivo models and clinical trials are essential to validate and extend these findings.

## Supporting information

S1 Fig Molecular docking findings showing the ligands binding to the core target proteins. (A) KQV-6NJS, (B) Calcitriol-6NJS, (C) ONJ-7LT0, (D) Calcitriol-7LT0, (E) UOW-7NR9, (F) Calcitriol-7NR9, (G) 1LT-4JPS, (H) Calcitriol-4JPS.
(TIF)

S2 Fig Molecular docking findings revealing the hydrophobic interactions between ligands and the core target proteins. Hydrophobic interactions are shown as red opposite arcs. Hydrogen bonds are indicated by green dashed lines. (A) KQV-6NJS, (B) Calcitriol-6NJS, (C) ONJ-7LT0, (D) Calcitriol-7LT0, (E) UOW-7NR9, (F) Calcitriol-7NR9, (G) 1LT-4JPS, (H) Calcitriol-4JPS.
(TIF)

S1 File Supporting information for Fig 7. (PDF)

S2 File Supporting information for Fig 8E. (PDF)

S1 Table Related genes of calcitriol, ERS, and T2DM. Abbreviations: T2DM: type 2 diabetes mellitus; ERS: endoplasmic reticulum stress.
(XLS)

S2 Table Topological analyses of the intersected target genes. (XLSX)

S3 Table Biological processes of the core target genes. (XLSX)

S4 Table Cellular component of the core target genes. (XLSX)

S5 Table Molecular function of the core target genes. (XLSX)

S6 Table KEGG pathways of the core target genes. Abbreviations: KEGG: Kyoto Encyclopedia of Genes and Genomes.
(XLSX)

S7 Table Classified KEGG pathways of the core target genes. Abbreviations: KEGG: Kyoto Encyclopedia of Genes and Genomes.
(XLSX)
