# Supplementary figures and images for: Unveiling the mechanism of calcitriol in treating type 2 diabetes mellitus: A combined network pharmacology and in vitro approach targeting ERS-related pathways
Source: PLoS One. 2026 Apr 16;21(4):e0347246. doi: 10.1371/journal.pone.0347246 (PMC13086306; doi:10.1371/journal.pone.0347246)

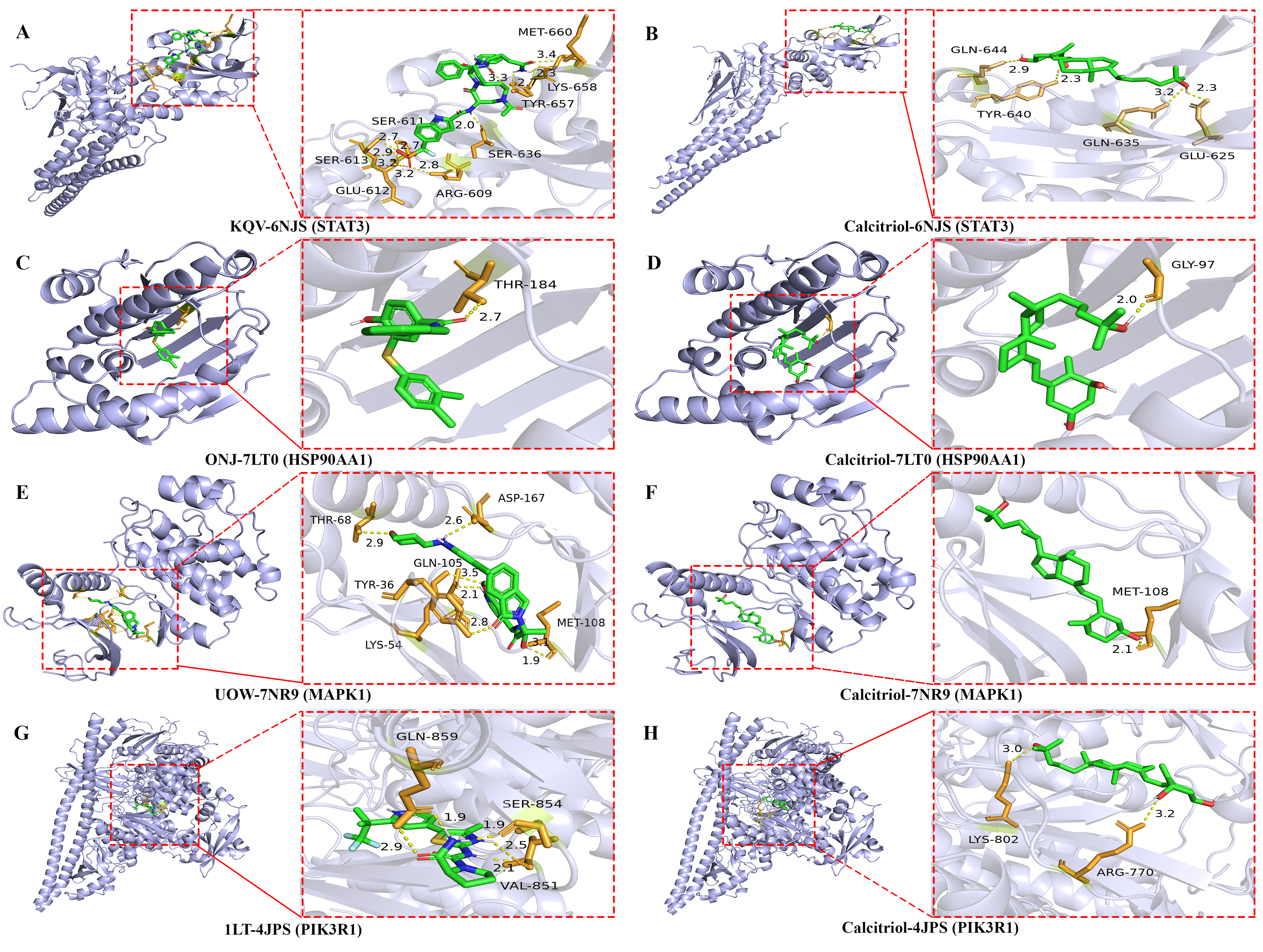

Supplement: S1 Fig — (A) KQV-6NJS, (B) Calcitriol-6NJS, (C) ONJ-7LT0, (D) Calcitriol-7LT0, (E) UOW-7NR9, (F) Calcitriol-7NR9, (G) 1LT-4JPS, (H) Calcitriol-4JPS. (TIF) [file pone.0347246.s001.tif]

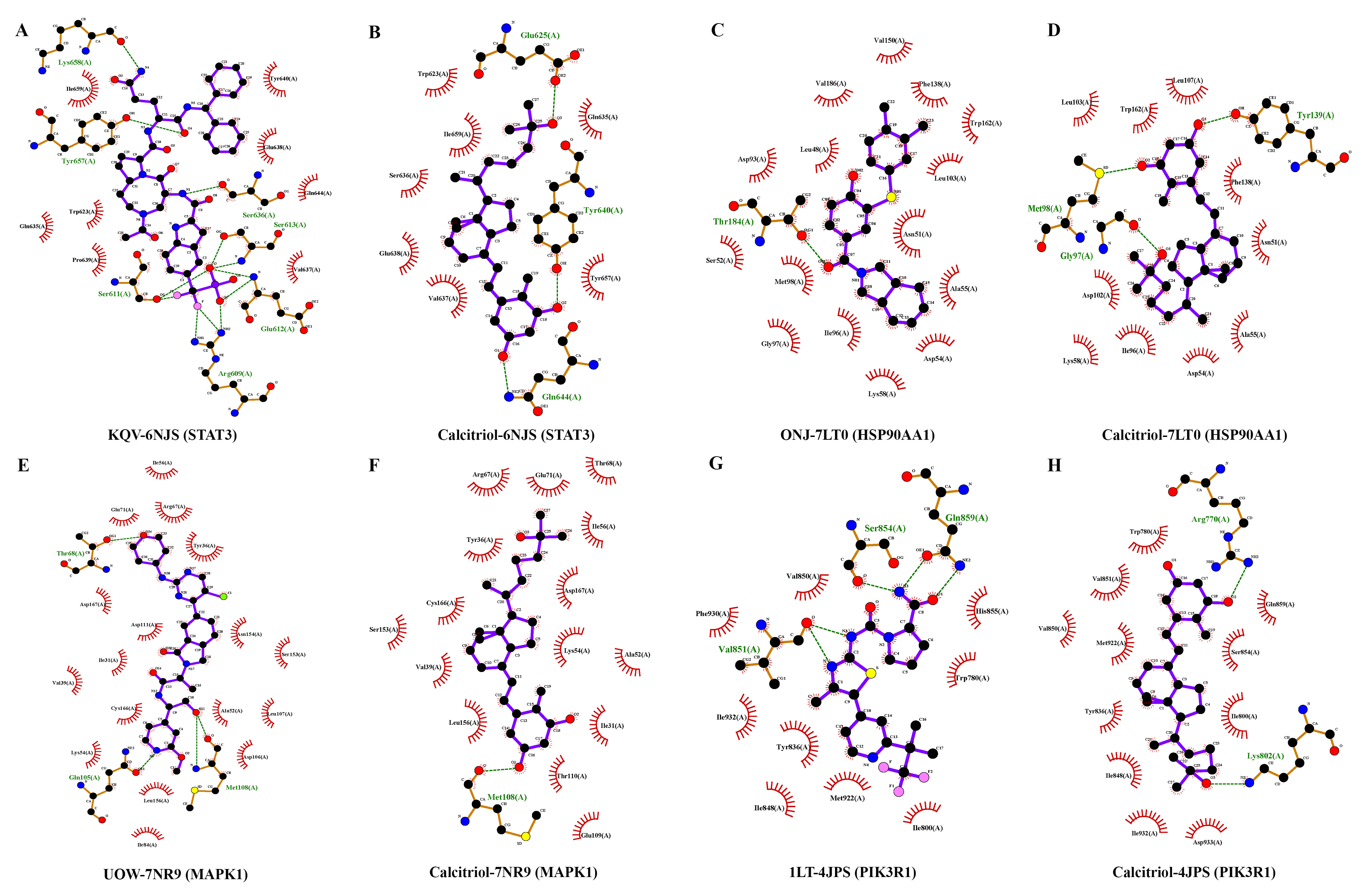

Supplement: S2 Fig — Hydrophobic interactions are shown as red opposite arcs. Hydrogen bonds are indicated by green dashed lines. (A) KQV-6NJS, (B) Calcitriol-6NJS, (C) ONJ-7LT0, (D) Calcitriol-7LT0, (E) UOW-7NR9, (F) Calcitriol-7NR9, (G) 1LT-4JPS, (H) Calcitriol-4JPS. (TIF) [file pone.0347246.s002.tif]

Raw Images for Figure 7

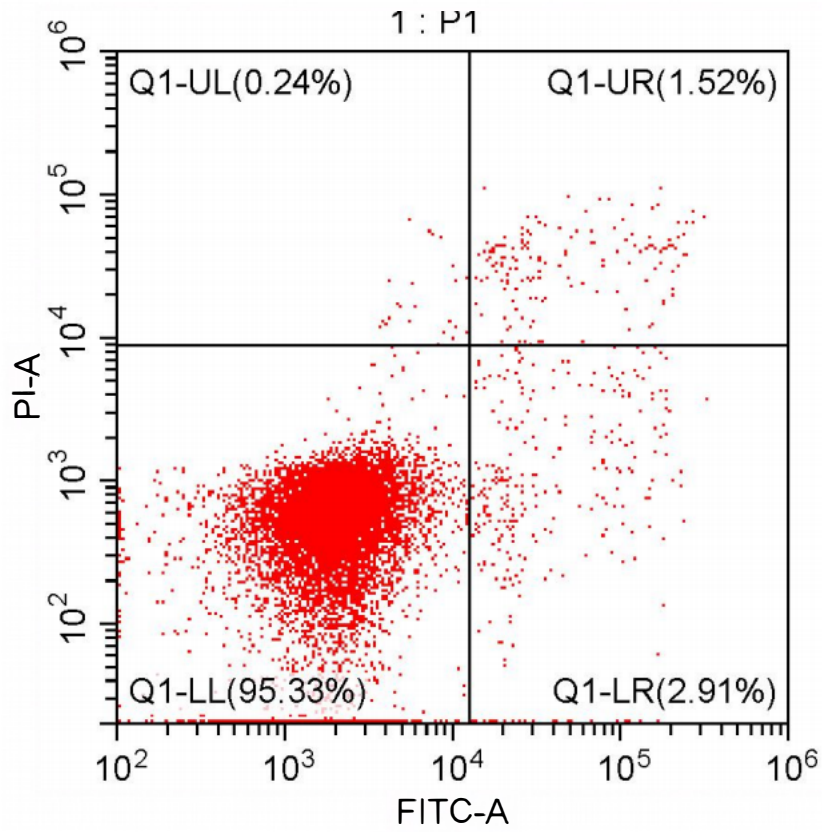

NG-1

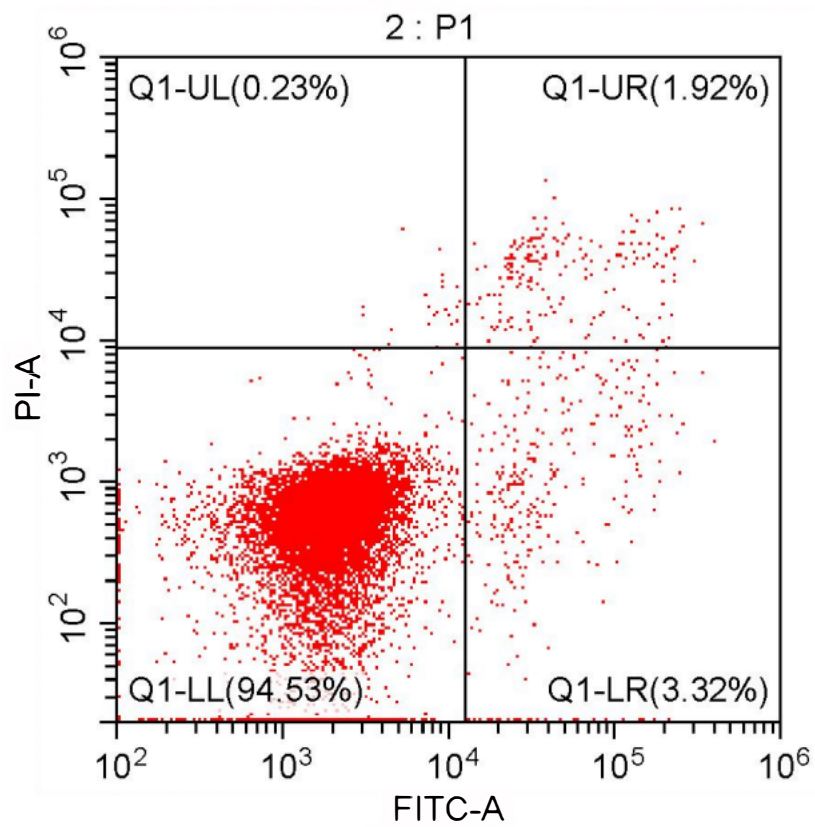

NG-2

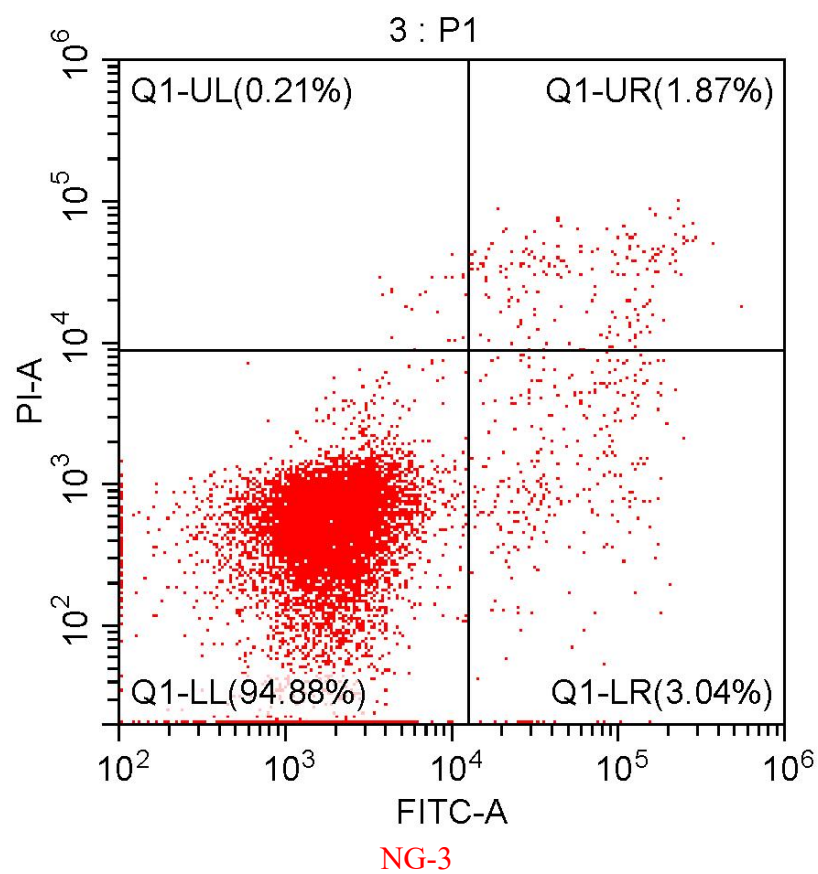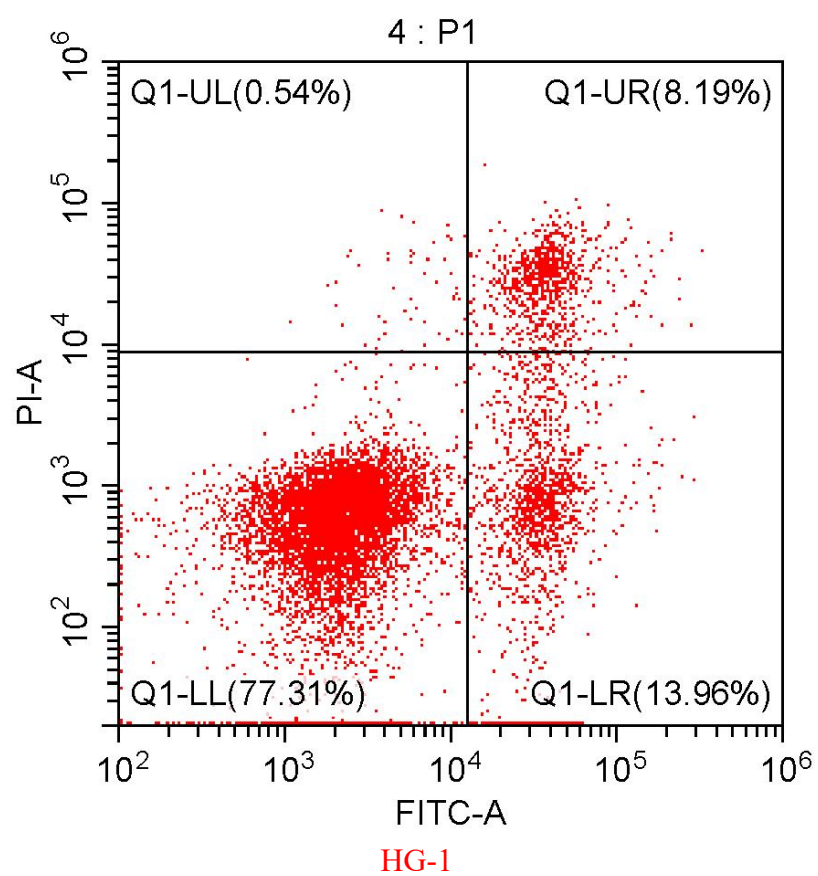

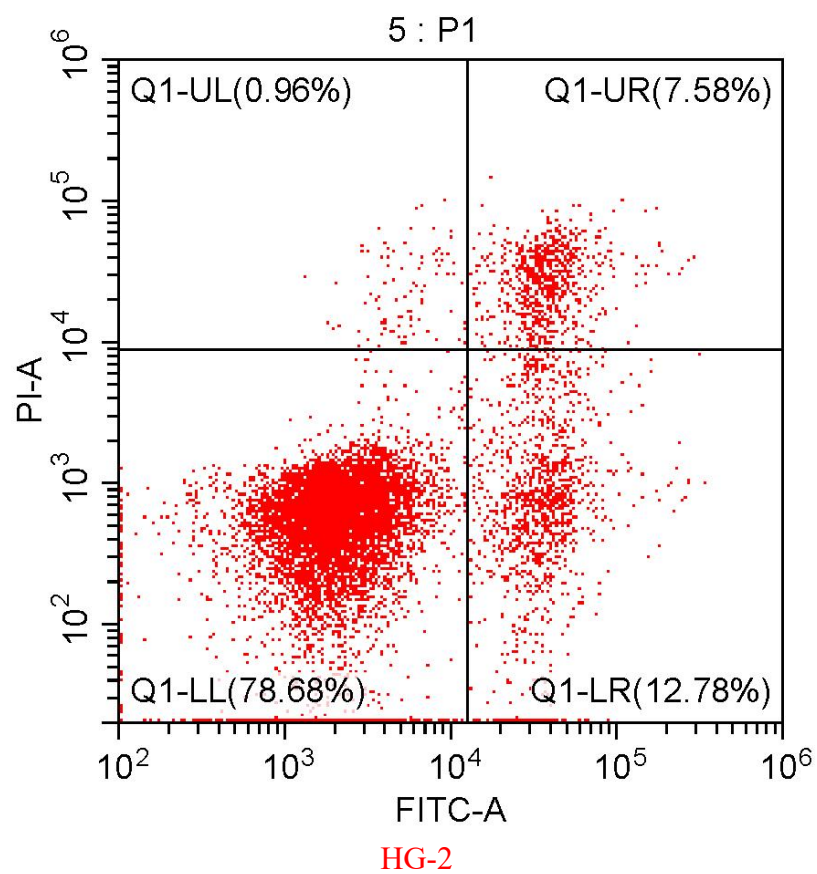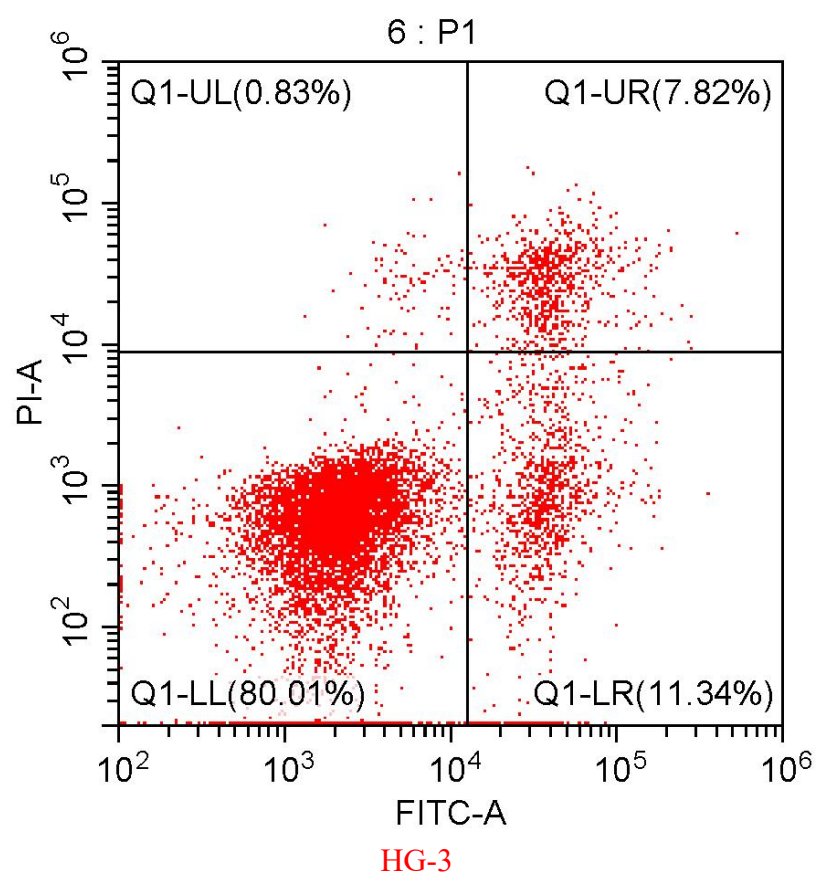

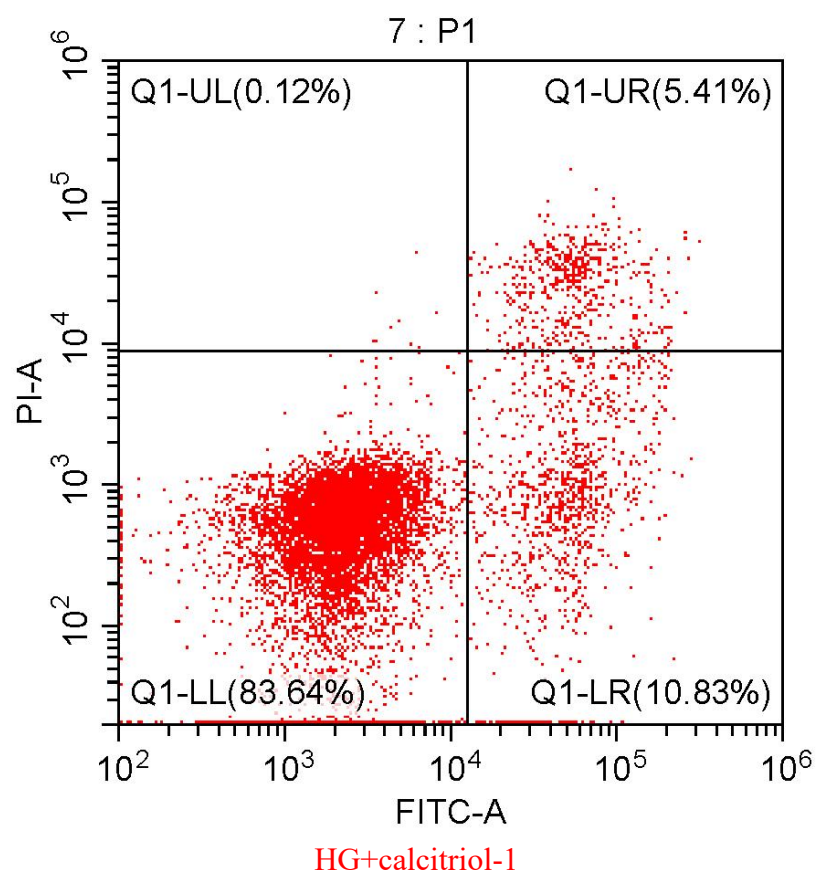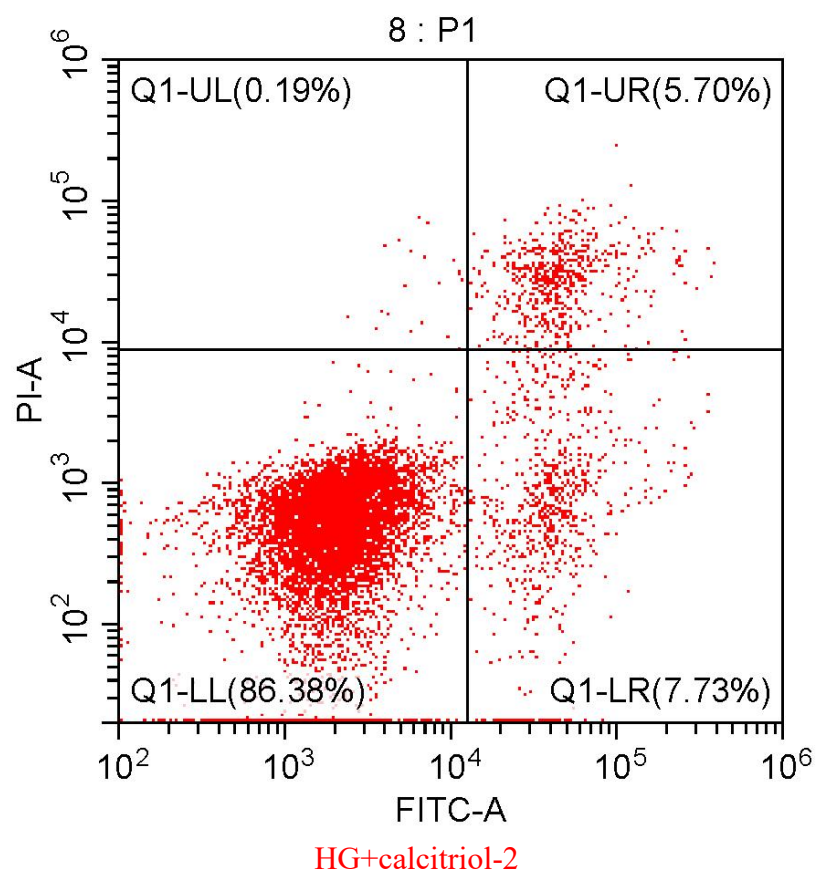

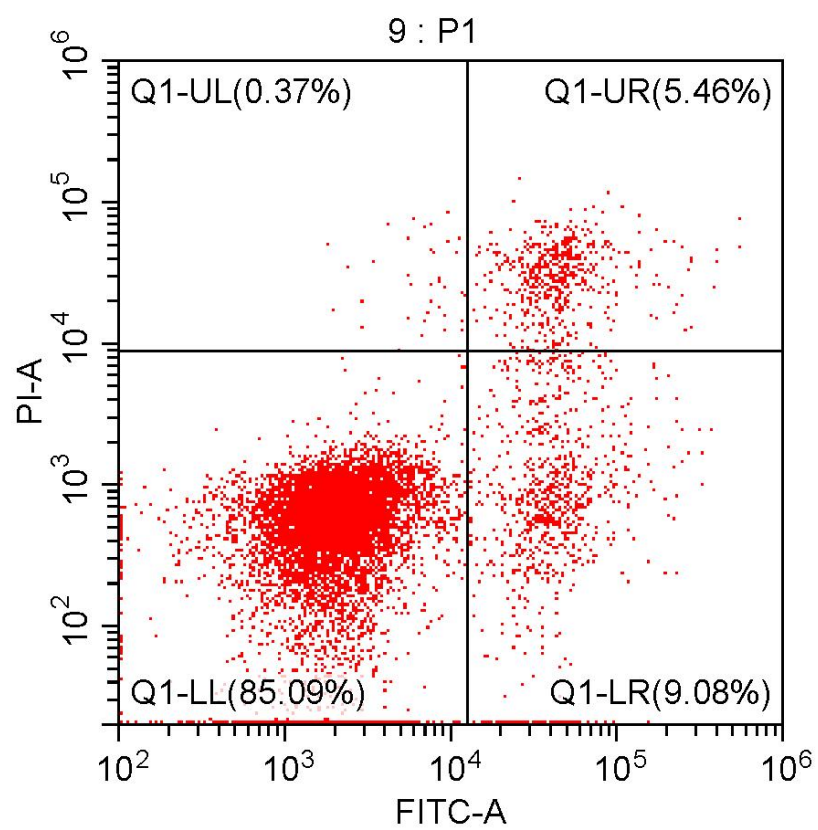

HG+calcitriol-3

Supplement: S1 File — (PDF) [file pone.0347246.s003.pdf]

# Raw Images for Figure 8E ( 2 )

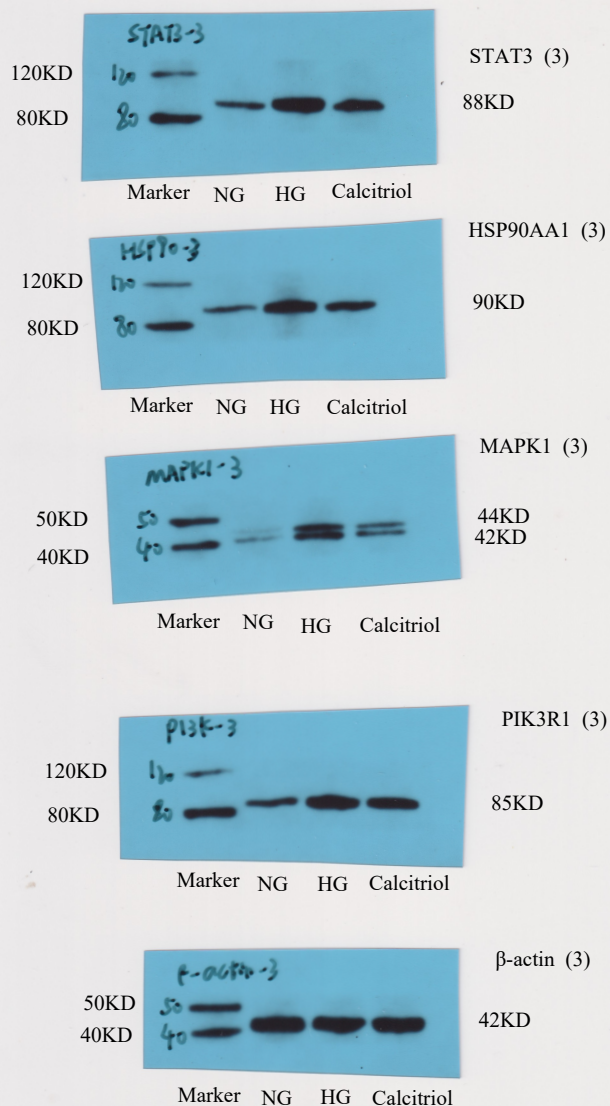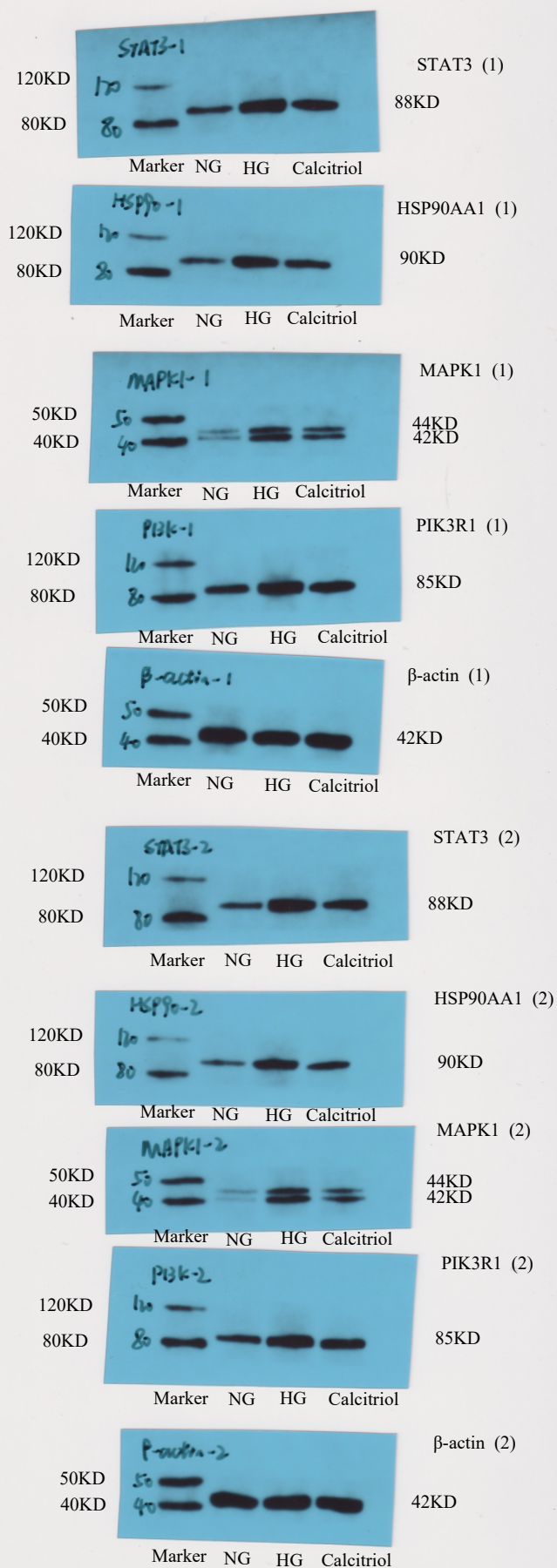

Supplement: S2 File — (PDF) [file pone.0347246.s004.pdf]
